# Identifying Clinicopathological Risk Factors of the Regional Lymph Node Metastasis in Patients with T_1-2_ Mucinous Breast Cancer: A Population-Based Study

**DOI:** 10.1155/2021/3866907

**Published:** 2021-07-09

**Authors:** Yu Min, Xiaoyuan Wei, Hang Chen, Ke Xiang, Guobing Yin, Yang Feng

**Affiliations:** ^1^Department of Breast and Thyroid Surgery, The Second Affiliated Hospital of Chongqing Medical University, No. 74, Linjiang Rd., Yuzhong Dist., Chongqing 404100, China; ^2^Department of Cardiovascular Internal Medicine, The Second Affiliated Hospital of Chongqing Medical University, No. 74, Linjiang Rd., Yuzhong Dist., Chongqing 404100, China

## Abstract

**Background:**

Pure mucinous breast cancer (PMBC) has a better prognosis than other types of invasive breast cancer. However, regional lymph node metastasis (LNM) might reverse this outcome. We aim to determine the independent predictive factors for regional LNM and further develop a nomogram model for clinical practice.

**Method:**

Data of PMBC patients from the Surveillance, Epidemiology, and End Results (SEER) program between Jan 2010 and Dec 2015 were retrospectively reviewed. Univariate and multivariate logistic regression analyses were used to determine the risk factors for LNM in T_1-2_ MBC. The nomogram was constructed and further evaluated by an internal validation cohort. The receiver operating characteristic (ROC) curves, decision curve analysis (DCA), and calibration curves were performed to evaluate the accuracy of this model.

**Result:**

Five variables, including age, race, tumor size, grade, and breast subtype, were identified to be significantly associated with regional LNM in female patients with T_1-2_ PMBC. A nomogram was successfully established with a favorable concordance index (C-index) of 0.780, supported by an internal validation cohort with a C-index of 0.767.

**Conclusion:**

A nomogram for predicting regional LNM in female patients with T_1-2_ PMBC was successfully established and validated via an internal cohort. This visualized model would assist surgeons to make appropriate clinical decisions in the management of primary PMBC, especially in terms of whether axillary lymph node dissection (ALND) is warranted.

## 1. Introduction

Nowadays, the prevalence of breast cancer is rapidly increasing and has become the leading cancer with the highest incidence rate for women worldwide, according to the latest report from the American Cancer Society [1]. Compared with not otherwise specified (NOS) invasive ductal carcinoma (IDC), pure mucinous breast cancer (PMBC) is a pathologically and genetically distinct mammary neoplasm, representing 1–4% breast cancer and containing a relatively good prognosis [2–5]. However, as one pivotal prognostic determinant, axillary lymph node metastasis (ALNM) was still determined to be negatively correlated with the long-term survival of PMBC [6, 7]. Therefore, the regional lymph node status is crucial for clinicians to make appropriate treatment decisions for this special type of breast cancer.

Undoubtedly, sentinel lymph node biopsy (SLNB) was regarded as an efficacy intraoperative strategy for predicting the potential ALNM in patients with clinically node-negative (cN0) breast cancer. It could not only guide the surgeons to measure the necessity of axillary lymph node dissection (ALND) but further help to reduce the postoperative complications. Moreover, the feasibility and accuracy of SLNB results after neoadjuvant chemotherapy (NAC) in patients with initial node involvement were determined in recent meta-analysis [8]. And the SLNB was even sufficient and reliable for patients with initial biopsy-proven node-positive breast cancer but converted to negative after NAC [9]. However, the false-negative (FN) result of SLNB was inevitable, especially in the presence of a small primary tumor with a single nodal metastasis [10, 11]. It was deemed to be a potential problem in the completeness of surgical dissection procedures. The FN rate, accounting for approximately 2%–27% in different studies [8, 10], was significantly associated with the numbers of examined sentinel lymph nodes. Notably, Moo et al. conducted that micrometastases or isolated tumor cells (ITCs) after NAC were not an indicator of exempting for additional ALND, even when not detected on intraoperative SLNB [12]. Thus, weighing the potential risk of ALNM which was not intraoperatively detected via the SLNB, more indicators were demanded for comprehensively predicting the regional lymph node metastasis (LNM) in patients with breast cancer.

With the popularization of breast mammography and ultrasound, an increasing number of female patients with small primary breast cancer were screened out and clinically diagnosed. Despite the relatively small primary tumor focus (T_1-2_), defined as a maximum diameter less than or equal to 50 mm, considerable patients still suffered from regional metastasis, bone metastasis, and even visceral metastases at initial diagnosis [13]. Moreover, only a small number of listed studies are focused on investigating the risk factors for predicting the prognosis in patients with PMBC [6, 7, 14].

In the present study, we hereby aim to explore the clinicopathological risk factors of promoting regional LNM and further construct a new nomogram for predicting this event in female patients with T_1-2_ primary PMBC, which would assist clinicians to preoperatively identify high-risk patients and make better individualized surgical decisions.

## 2. Materials and Methods

### 2.1. Data Source

The data we analyzed were extracted from the Surveillance, Epidemiology, and End Results (SEER) database, derived from the 18 cancer registries across the United States of America (USA), covering approximately 28% of incident cases of the whole country (http://seer.cancer.gov) and included various ethnic groups.

Patients who met the following criteria were included:

1. Female patients between the age of 20 and 84 years.
2. Diagnosis year between 2010 and 2015.
3. The diagnosis of PMBC was confirmed by histopathology, identified based on the International Classification of Diseases for Oncology, 3rd Revision (ICD-O-3) codes (8480/3).
4. T_1-2_ stage breast cancer derived from adjusted AJCC staging system, 7th edition.

Patients with no regional node examined, presence of distant metastasis, or coexisting with one or more cancers were excluded during the study period.

### 2.2. Data Analysis

After excluding unqualified patients, there were 3,111 patients with PMBC in the SEER program enrolled in this study. The patients diagnosed between 2010 and 2013 were designed as the training group and patients diagnosed between 2014 and 2015 were designed as the validating group, respectively. The following clinicopathological characteristics were collected and transformed into categorical variables: age, race, gender, laterality, grade, location, size, histological type, and the number of regional nodes examined and positive nodes.

Univariate and multivariate regression analyses performed by IBM SPSS (version 25.0) were used to identify the independent risk factors in patients. A two-tailed *p*-value of <0.05 was defined as the criterion for variable deletion when performing backward stepwise selection. The development and validation of nomograms were based on the results of the multivariate logistic regression analysis using the rms package of the R software (R Foundation, Vienna, Austria, version 3.5.2, http://www.r-project.org). Harrell's C-index, which is equivalent to the area under the ROC curve, is calculated to assess the discrimination performance of the present nomograms.

## 3. Result

### 3.1. Baseline Characteristics

A total of 3,111 female patients with PMBC who met the inclusion criteria were enrolled in this study. All the patients were histologically diagnosed with primary PMBC and the maximum diameter of the tumor was less than or equals to 50 mm (T_1-2_). Overall, the regional LNM was identified in 240 (7.71%) cases of the initial cohort, including 152 (8.06%) of the training group and 88 (7.18%) of the validation group, respectively. Besides, among the training and validating cohorts, a majority of patients were white (76.09% and 73.31%, resp.). Moreover, luminal A (94.09%) was the predominant tumor subtype of PMBC in this study, compared with other tumor subtypes including luminal B (4.73%), TNBC (0.55%), and HER2 enriched (0.64%). The specific demographic and clinical characteristics of the patients in the training and validation datasets were summarized in Table 1.

### 3.2. Univariate and Multivariate Analyses of the Risk of Regional LNM

Attempting to determine the predictive factors of regional LNM in female patients with T_1-2_ PMBC, eight variables including age, race, tumor size, location, differentiation grade, laterality, and tumor subtype were initially analyzed in univariate analysis (Table 2). Five variables which were significantly different (age, grade, size, race, and tumor subtype) were obtained by univariate analysis (all *p* < 0.05) and were analyzed by multivariate logistic regression analysis. The risk factors which were significantly associated with regional LNM were as follows: aged under 45 years (*p*=0.001), tumor size (5 mm < largest diameter ≤ 10 mm, odds ratio (OR) = 1.04, 95% confidence interval (CI): 0.19–5.54; 10 mm < largest diameter ≤20 mm, OR = 6.12, 95% CI: 1.44–25.98; 20 mm < largest diameter ≤50 mm, OR = 15.29, 95% CI: 3.62–64.49; *p* < 0.001), black race (OR = 1.68, 95% CI: 1.05–2.69; *p*=0.004), and grade (II, OR = 1.94, 95% CI: 1.34–2.80; III, OR = 1.98, 95% CI: 0.95–4.15; *p*=0.001). In addition, TNBC patients had a higher risk of regional LNM compared with luminal A type of patients (OR = 3.06, 95% CI: 0.73–12.86; *p*=0.029). However, there were neither significant differences in the tumor location nor laterality for predicting the risk of regional LNM (*p*_1_=0.74, *p*_2_=0.80, resp.).

### 3.3. Predictive Nomogram Construction and Validation

**Figure 1:**
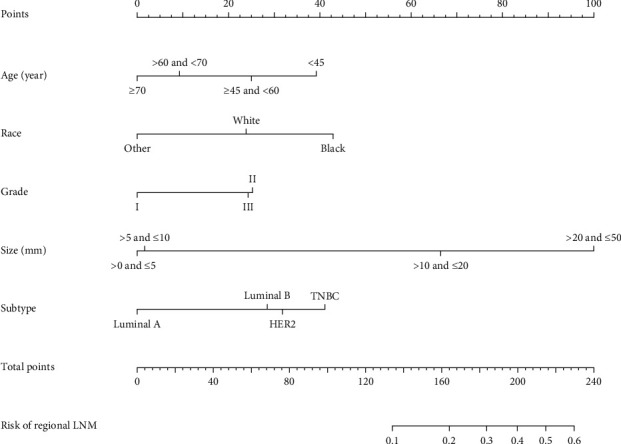
Nomogram for predicting the regional lymph node metastasis (LNM) in female patients with T1-2 pure mucinous breast cancer (PMBC). Note: others: defined as the Asian/Pacific Islander and American Indian/Alaska Native; grade I: well differentiated, grade II: moderately differentiated, and grade III: poorly differentiated; stage: derived from the adjusted AJCC 7th guideline. LNM: lymph node metastasis; HR: hormone receptor; TNBC: triple-negative breast cancer; HER2: human epidermal growth factor receptor-2.

**Figure 2:**
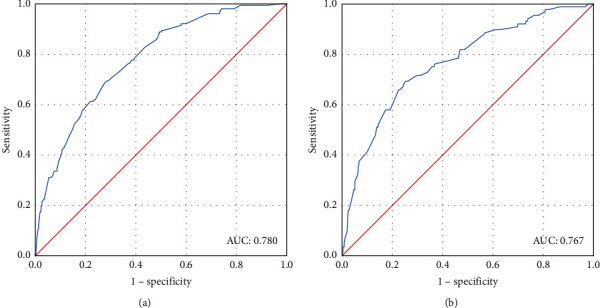
(a) The receiver operating characteristics (ROC) curve and area under the ROC curve (AUC) in the training cohort. (b) The receiver operating characteristics (ROC) curve and area under the ROC curve (AUC) in the validation cohort.

Based on the analysis results of multivariate logistic regression, the independent variables including age, race, grade, tumor size, and tumor subtype were screened out for establishing a visualized nomogram to predict regional LNM in female patients with primary T_1-2_ PMBC (Figure 1). The concordance index (C-index), which was equivalent to the AUC (area under the curve) of ROC, was 0.783 (Figure 2(a)). Moreover, to validate the utility of our nomogram, an internal validation cohort by using data (1,225 cases) between 2014 and 2015 from the SEER program was subsequently constructed, rendering a similar C-index of 0.767 (Figure 2(b)), which indicated an optimistic outcome of our nomogram in predicting the regional lymph node involvement in female patients with primary T_1-2_ PMBC.

**Figure 3:**
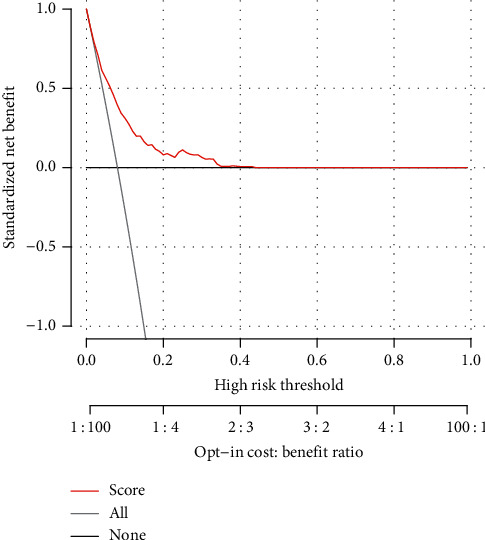
Decision curve analysis for regional lymph node metastasis (LNM) in female patients with T1-2 pure mucinous breast cancer (EI-score) in the training cohort and internal cohort. The decision curve analysis graphically shows the clinical usefulness of the EI-score based on a continuum of potential thresholds for regional LNM and the net benefit of using the EI-score to stratify patients (*y*-axis). Net benefit = (true positives/*N*)−(false positives/*N*)*∗*(weighting factor). Weighting factor = Threshold probability/(1-threshold probability).

**Figure 4:**
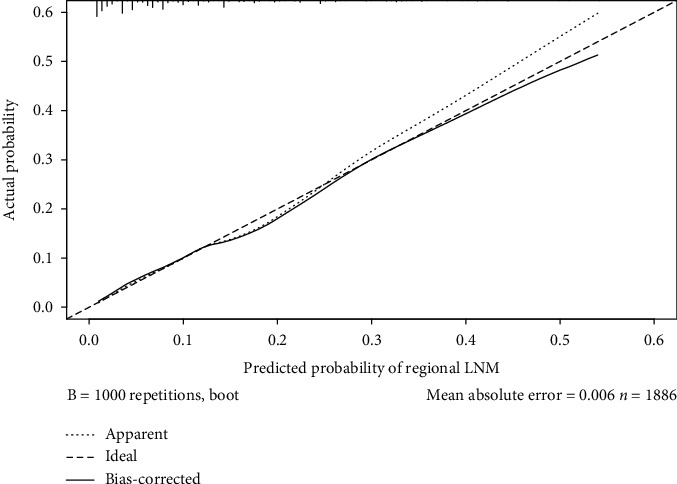
Calibration curves of the nomogram of training cohort for predicting regional lymph node metastasis (LNM) in female patients with T1-2 pure mucinous breast cancer (bootstrap 1000 repetitions). The *x*-axis represents the predicted regional LNM. The *y*-axis represents the actual LNM. The diagonal dotted line stands for a perfect prediction using an ideal model. The solid line represented the performance of the nomogram, of which the closer fit to the diagonal dotted line represents the better prediction of the nomogram we constructed.

To further evaluate the predictive ability of the nomogram, a decision curve analysis (DCA) was performed both in training and in internal datasets. The standardized net benefits of the models were comparable, and there was a significant overlap between these models. Namely, the DCA showed that the prediction ability of the nomogram was more effective than a treat-none or treat-all strategy when the threshold probability ranged from 0.05 to 0.5 (Figure 3). Furthermore, a calibration curve of the regional LNM risk nomogram in female patients with PMBC was also displayed. The result suggested a great agreement in the training data set, with a mean absolute error = 0.006 (Figure 4).

## 4. Discussion

Currently, breast cancer is the leading malignancy among women, with the highest incidence rate worldwide, especially in the United States (accounting for approximately 30% of new cases) [1]. Although great advances have been made in therapeutic modalities, including but not limited to surgical techniques, adjuvant chemotherapy, radiotherapy, and even immunotherapy for delaying disease progression and improving the long-term prognosis, early diagnosis, and systemic preoperative evaluation remained to be the crucially important steps for these patients.

During the past years, a lot of research has been done on determining the risk factors for lymph node involvement and survival in patients with different subtypes of breast cancer or coexisting with distant metastasis at initial diagnosis [13, 15–19]. As IDC and invasive lobular carcinoma (ILC) accounted for the vast majority of cases, the clinicopathological characteristics of these two types of breast cancer were the main object of intensive research. For instance, Wang et al. established a nomogram for predicting the prognosis of female patients with breast cancer and bone metastasis at presentation [19] and Cui et al. [15] established a nomogram for predicting the LNM in TNBC patients. On the contrary, PMBC, as a rare histologic type of mammary neoplasm, accounting for 1–4% of all breast cancers, has rarely been investigated and usually was classified as “others” group in several studies [14, 16, 19, 20]. Recently, increasing attention has been paid to the treatment modalities, especially the necessity of adjuvant chemotherapy, radiotherapy, and anti-HER2 therapy for this kind of malignancy [21–25]. Notably, the role of chemotherapy in PMBC was controversial. In two population-based studies from the SEER database and Korean Breast Cancer Registry [21, 25], patients with PMBC could not benefit from chemotherapy during long-time survival and they further concluded that these patients could be exempt from chemotherapy. However, in one most recent published literature, Gao [26] conducted that early-stage HR (hormone receptor) positive PMBC patients could benefit from the adjuvant chemotherapy, especially in terms of having a better overall survival (OS) when compared with nonchemotherapy patients (*p* < 0.001). Although several previous studies have determined that positive lymph node status was the most important prognostic factor which could affect and worsen the prognosis [6, 7], there was still a lack of an effective predictive model to evaluate the risk of regional LNM and further guide whether the ALND was appropriate during the surgical intervention in patients with PMBC.

In the present study, to the best of our knowledge, this was the first validated nomogram for predicting regional LNM in female patients with T_1-2_ PMBC based on the clinicopathological features. The regional LNM was determined in 7.71% of patients, which was lower than one study containing a larger sample size [6]. In the multivariate logistic regression analyses, age, tumor size, race, differentiation grade, and tumor subtype were significantly associated with regional LNM. Specifically, patients with younger age (<45 years), larger tumor size (>10 mm), black race, poor differentiation, HER2 enriched, or TNBC subtypes had a higher risk of regional LNM. These results were partially consistent with a previous study on evaluating the LNM in patients with different types of breast cancer. By contrast, tumor location and laterality were not regarded as predictive factors in the regional LNM of the PMBC. Interestingly, some studies covering large-scale populations reported that the primary tumor location was strongly correlated with positive axillary lymph nodes, particularly located in the nipple, central breast, or axillary tail [20, 27]. This different result might be attributed to the smaller study population and lower regional LNM rate in patients with PMBC when compared with IDC or other types of breast cancer [6].

In order to build a more convenient and visualized predictive model for clinical practice with the variables we determined above, a novel nomogram was successfully established. The risk of positive lymph nodes predicted by our nomogram ranged from 0.1 to 0.6. Besides, the C-index, which was in accordance with the AUC value in ROC of our nomogram was much higher than 0.70. It therefore indicates that our nomogram has sufficient discrimination ability. Moreover, the DCA results show that the nomogram we developed has a good clinical practical value. To further evaluate the feasibility of our nomogram, an internal validation cohort consisting of 1,225 female PMBC patients diagnosed between 2014 and 2015 years in the SEER database was performed. As expected, the predictive ability in the validation group was satisfied with a C-index of 0.767. Referencing similar work on predicting LNM in patients with different subtypes of breast cancer, our study took it a step further. For instance, while the study population in one nomogram constructed by Cui et al. [15] for predicting the LNM in TNBC patients was larger than ours, the C-index of the training set was only 0.684. Besides the nomogram for predicting the LNM in T_1_ breast cancer developed by Zhao et al. [20], the C-index of the training group and validation group achieved 0.733 and 0.741, respectively, which were still weaker than ours. Thus, these results confirmed the utility of our nomogram in predicting regional LNM in patients with T_1-2_ PMBC. Additionally, the patients were stratified into different risk subgroups according to the nomogram, and a higher prevalence of LNM was observed in high-risk subgroups. Nowadays, the comprehensive treatment modalities of PMBC were still controversial but worth further exploration [21, 23, 26]. This nomogram combined with other preoperative indicators [28] could not only help surgeons to decide whether ALND was appropriate for patients with T_1-2_ PMBC but also offered an alternative way for stratification which could assist to select patients for adjuvant therapy.

Nonetheless, there were some limitations in our study, which we needed to clarify and address in the following research. First, this is a retrospective cohort study which may inevitably lead to some selected bias. Second, while the sample size of female patients with PMBC in our study was considered proper, yet it remains smaller than several studies on assessing the risk factors of regional LNM or long-term survival in patients with breast cancer [13, 14, 20]. Third, the vast majority of the study race is white (74.99%). For this reason, whether this nomogram could apply to other patients of different races and regions needs further exploration and external validation. Further prospective randomized controlled studies are needed to obtain more detailed strategies on the treatment of PMBC.

## 5. Conclusion

In summary, five clinical risk factors including age, race, tumor size, grade, and breast subtype, were identified to be significantly associated with regional LNM in female patients with T_1-2_ PMBC. And a novel nomogram for predicting regional LNM in female patients with T_1-2_ PMBC was successfully established, supported by the internal validation datasets. Our model could not only provide a more accurate reference for surgeons to better identify individuals at risk for regional LNM preoperatively but also help to make appropriate clinical decisions in the management of primary T_1-2_ PMBC.

## Figures and Tables

**Table 1:** Clinicopathological characteristics of female patients with T_1-2_ pure mucinous breast cancer.

Variables	Subgroup	No. (%) of patients		
		Initial cohort (*n* = 3,111)	Training cohort (*n* = 1,886)	Validating cohort (*n* = 1,225)
Age (year)	<45	286 (9.19)	179 (9.49)	107 (8.73)
	≥45 and <60	790 (25.39)	482 (25.56)	308 (25.14)
	≥60 and <70	942 (30.28)	542 (28.74)	400 (32.65)
	≥70	1,093 (35.13)	683 (36.21)	410 (33.47)

Race	White	2,333 (74.99)	1,435 (76.09)	898 (73.31)
	Black	378 (12.15)	215 (11.40)	163 (13.31)
	^**※**^Others	400 (12.86)	236 (12.51)	164 (13.39)

Location	^**¶**^Central	219 (7.04)	150 (7.95)	69 (5.63)
	Upper	1,329 (42.72)	802 (42.52)	527 (43.02)
	Lower	680 (21.86)	425 (22.53)	255 (20.82)
	Axillary tail	13 (0.42)	10 (0.53)	3 (0.24)
	Overlapping	870 (27.97)	499 (26.46)	371 (30.29)

^＆^Grade	I	1,897 (60.98)	1,163 (61.66)	734 (59.92)
	II	1,107 (35.58)	649 (34.41)	458 (37.39)
	III	107 (3.44)	74 (3.92)	33 (2.69)

Laterality	Right	1,487 (47.80)	888 (47.08)	599 (48.90)
	Left	1,624 (52.20)	998 (52.92)	626 (51.10)

^￥^Stage	I	2,120 (68.15)	1,294 (68.61)	826 (67.43)
	II	962 (30.92)	575 (30.49)	387 (31.59)
	III	29 (0.93)	17 (0.90)	12 (0.98)

Tumor size (mm)	>0 and ≤5	225 (7.23)	142 (7.53)	83 (6.78)
	>5 and ≤10	663 (2.12)	409 (21.69)	254 (20.73)
	>10 and ≤20	1,290 (41.47)	784 (41.57)	506 (41.31)
	>20 and ≤50	933 (29.99)	551 (29.21)	382 (31.18)

HR status	Positive	3,074 (98.81)	1,858 (98.51)	1,216(99.27)
	Negative	37 (1.19)	28 (1.48)	9 (0.73)

HER2 status	Positive	167 (5.37)	114 (6.04)	53 (4.33)
	Negative	2,944 (94.63)	1,772 (93.95)	1,172 (95.67)

Tumor subtype	Luminal A	2,927 (94.09)	1,761 (93.37)	1,166 (95.18)
	Luminal B	147 (4.73)	97 (5.14)	50 (4.08)
	TNBC	17 (0.55)	11 (0.58)	6 (0.49)
	HER2 enrich	20 (0.64)	17 (0.90)	3 (0.24)

Regional LNM	Yes	240 (7.71)	152 (8.06)	88 (7.18)
	No	2,871 (92.29)	1,734 (91.94)	1,137 (92.82)

Notes: ^※^Others: defined as the Asian/Pacific Islander and American Indian/Alaska Native; ^¶^central: central portion of breast combined with nipple; ^＆^grade I: well differentiated, grade II: moderately differentiated, and grade III: poor differentiated; ^￥^stage: derived from the AJCC 7th guideline. Cervical LNM: central and lateral lymph node metastasis; HR: hormone receptor; TNBC: triple-negative breast cancer; HER2: human epidermal growth factor receptor-2.

**Table 2:** Univariate and multivariate logistic regression analyses of predictive factors associated with regional LNM in patients with T_1-2_ pure mucinous breast cancer.

Variables	Subgroup	Univariable		Multivariable	
		Hazard ratio	*p*	Hazard ratio	*p*
Age	<45	Reference	**<0.001**	Reference	**0.001**
	≥45 and <60	0.69 (0.42–1.13)		0.68 (0.40–1.14)	
	≥60 and <70	0.37 (0.21–0.63)		0.44 (0.25–0.77)	
	≥70	0.28 (0.16–0.48)		0.34 (0.19–0.60)	

Tumor size (mm)	>0 and ≤5	Reference	**<0.001**	Reference	**<0.001**
	>5 and ≤10	0.86 (0.16–4.51)		1.04 (0.19–5.54)	
	>10 and ≤20	5.17 (1.24–21.48)		6.12 (1.44–25.98)	
	>20 and ≤50	13.84 (3.36–56.93)		15.29 (3.62–64.49)	

Race	White	Reference	**0.008**	Reference	**0.004**
	Black	1.91 (1.23–2.96)		1.68 (1.05–2.69)	
	^**※**^Others	0.83 (0.47–1.45)		0.52 (0.29–0.94)	

Location	^**¶**^Central	Reference	0.749	—	—
	Upper	1.11 (0.57–2.16)		—	—
	Lower	1.06 (0.52–2.16)		—	—
	Axillary tail	3.15 (0.59–16.72)		—	—
	Overlapping	1.13 (0.56–2.26)		—	—

^＆^Grade	I	Reference	**<0.001**	Reference	**0.001**
	II	2.50 (1.76–3.55)		1.94 (1.34–2.80)	
	III	3.49 (1.79–6.83)		1.98 (0.95–4.15)	

Laterality	Right	Reference	0.808	—	—
	Left	0.96 (0.68–1.33)		—	—

Tumor subtype	Luminal A	Reference	**<0.001**	Reference	**0.029**
	Luminal B	2.88 (1.67–4.95)		2.17 (1.19–3.96)	
	TNBC	4.74 (1.24–18.09)		3.06 (0.73–12.86)	
	HER2 enriched	1.68 (0.38–7.45)		2.38 (0.46–12.09)	

Notes: ^※^Others: defined as the Asian/Pacific Islander and American Indian/Alaska Native. Bold values indicate statistical significance (*p* < 0.05). ^¶^Central: central portion of breast combined with nipple; ^＆^grade I: well differentiated, grade II: moderately differentiated, and grade III: poorly differentiated. LNM: lymph node metastasis; TNBC: triple-negative breast cancer; Her-2: human epidermal growth factor receptor-2.

## Data Availability

The datasets generated during and/or analyzed during the current study are available from the corresponding author upon reasonable request.

## Abbreviations

PMBC: Pure mucinous breast cancer
LNM: Lymph node metastasis
SEER: Surveillance, Epidemiology, and End Results
ALND: Axillary lymph node dissection
IDC: Invasive ductal carcinoma
cN0: Clinically node-negative
ITCs: Isolated tumor cells
NAC: Neoadjuvant chemotherapy
ILC: Invasive lobular carcinoma
OS: Overall survival
TNBC: Triple-negative breast cancer
AUC: Area under the curve
ROC: Receiver operating characteristic
DCA: Decision curve analysis.
